# Supplementary material for: Kinesthetic illusions induced by muscle tendon vibration: The orientation of the vibration motor as a new methodological factor?
Source: PLoS One. 2025 Jun 9;20(6):e0325737. doi: 10.1371/journal.pone.0325737 (PMC12148079; doi:10.1371/journal.pone.0325737)
Supplement: Attachment — Submitted filename: Review Report.pdf [file pone.0325737.s003.pdf]

Overview:

Although this manuscript generally satisfies the seven criteria of PLOS ONE publication, there are concerns regarding important aspects of the study.

This work focuses on how the orientation of the vibration motor influences kinesthetic illusion, which could be somewhat useful to certain researchers and clinicians. However, before going through the Methods section, there is an immediate question coming to mind: Does flipping a motor's orientation affect the induced vibration?

As shown in the figure below (https://www.precisionmicrodrives.com/ab-014), when an eccentric rotating mass (ERM) vibration motor is placed on the skin, the induced vibration can be regarded as "bidirectional": one perpendicular to the skin (z-axis), and one perpendicular to the motor rotation axis while parallel to the skin (x-axis); no vibration is induced along the motor rotation axis (y axis). Most importantly, the orientation of the y-axis is irrelevant to the induced vibration: If the y-axis is rotated around the z-axis by 180°, x-axis is indeed flipped, but since the motor oscillates back and forth along the x-axis, vibration is unaffected. Similarly, vibration is independent of the direction of mass rotation (CW or CCW).

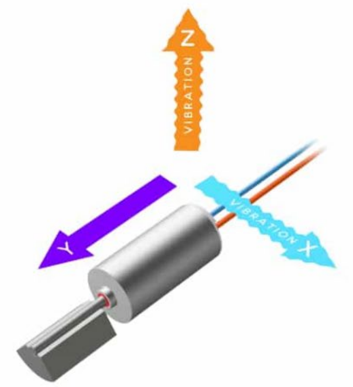

That said, a fundamental issue in the study design is that the induced vibrations in Figs 3a (proximodistal) and 3b (distoproximal) are essentially the same (so are Figs 3c and 3d), and the study is in fact comparing two types of vibrations (y-axis rotated around z-axis by 0° and 90°) instead of four (0°, 90°, 180°, 270°). While I agree flipping motor orientation should be controlled, this work treats it as a key variable without justification. The mechanism of the ERM vibration motor should be better explained, and the authors will need to demonstrate how and why flipping motor orientation could have affected the induced/perceived vibration, either by citation or measurement. Only then it is appropriate to investigate the impact on kinesthetic illusion.

Furthermore, it is unclear why data analysis is based on the median rather than the mean, which can be very misleading. For example, though no significance is reported, the results

in Table 2 seem to suggest that participants perceived different VAS speed/amplitude in all four conditions (e.g., 3.70 vs 4.00 vs 3.43 vs 4.30), which would be counter-intuitive given the mechanism of the ERM motor. If there are specific reasons to report median values, they must be stated. In the paper cited for the SKIP scoring system (ref. 19), the comparison was made based on mean values.

It is also confusing how movement perceived by the participant is "attributed by the evaluator to one of twelve possible perceptions at the wrist." There is no description of how this is performed, and if I understand correctly, the information mainly comes from verbal description, since the participant needs to "give specific details on the perceived movement." If this is the case, the results would be highly unreliable because normal participants are not trained to specify enough details for the evaluator to clearly categorize as many as 12 motions—the results in Table 3 would contain too much error for further analysis.

The authors are suggested to reexamine the study design and the collected data to perform solid analysis for the research question. I list below some of my suggestions for the textual content of this manuscript.

Specific comments:

1. [52] It is unclear why vibration should be abbreviated as VIB.
2. [57–59] I suppose "a few studies" refer to ref. 9–12, and in this case, the period after "retraining sensorimotor control" should be colon.
3. [65] Throughout the manuscript, the term "clear and precise" is used. While this appears to be two separate aspects, it is treated as a single property (at least indicated by the results). I suggest avoiding such ambiguity unless the authors have clear definitions of "clearness" and "precision" in the context of kinesthetic illusion. Also, avoid formats such as "clear & precise" or "clear/precise."
4. [70] The semicolon should be a colon.
5. [81–84] This text should be enhanced with discussion over the mechanism of vibration motor as well as how vibration direction is changed if the motor is rotated by 90°. Note that the rotational direction of the mass (equivalent to 180° rotation) is irrelevant to the induced vibration.
6. [95–96] This claim is inappropriate.
7. [99–102] The grammatic subject of this sentence should be "participants."
8. [117] "Then, …"
9. [142–146] The past tense suddenly changes to the present tense.
10. [168] Ref. 19 is the work of the corresponding author, so it is inappropriate to use the term "as recommended," as if the authors are citing an external source.

11. [191–196] How this is performed must be clarified.
12. [191–201] This should be part of the experimental procedure.
13. [Table 2] Report mean±STD.
14. [Table 3] Most motions have a frequency lower than 3%, which is just one or two trials and much more susceptible to error. If anything, only the last five rows should be shown, but the authors must prove the assessment was performed in a reliable way. For example, I don’t see how it is possible to separate the perception of extension/flexion from ulnar/radial deviation based on verbal description.
