# Supplementary material for: Kinesthetic illusions induced by muscle tendon vibration: The orientation of the vibration motor as a new methodological factor?
Source: PLoS One. 2025 Jun 9;20(6):e0325737. doi: 10.1371/journal.pone.0325737 (PMC12148079; doi:10.1371/journal.pone.0325737)
Supplement: Attachment — Submitted filename: Review Comments_r2.pdf [file pone.0325737.s005.pdf]

I would like to thank the authors for taking my previous comments into account, which must have taken a lot of time and effort. I am aware that it is somewhat unconventional (and seemingly unfair) that the request for revision increased tremendously after the first round of revision, but I was not among the first-round reviewers who could point out the critical issues at the early stage. After the second round of revision, the manuscript is now much improved.

Nevertheless, I must stick a bit more with the point that vibration is independent of rotational direction. The authors refuted in terms of intuition, theory, and experiment, which is highly appreciated but not entirely convincing. To sum up, the intuitive explanation is wrong, and the theoretical explanation fails to support the authors' claim (at least not by Refs 1 and 2); however, I cannot explain for the supporting evidence from the experimental measurements. Therefore, I believe it makes sense to present all results as they currently are, but the authors should make it clear that the reason for considering the rotational direction as a factor is because the accelerometer measurements suggest that the shaft orbit has a different shape (hence the vibration could be slightly different). Very importantly, this work is titled "The orientation of the vibration motor as a new methodological factor." So even if there is a slight difference in vibration between the rotational directions, it is due to some internal properties of the motor itself and is irrelevant to the orientation. Rotational direction serves as a control factor, and should not be the focus of discussion.

As I will address below, a few revisions might be based on wrong interpretation of vibration dynamics. The authors may decide whether they should be corrected or not, but please keep in mind that this is basic physics, and if explained incorrectly, some readers will doubt the results of the main experiments.

Intuitive Explanation

For scientific problems, one should never use intuition, otherwise one may end up with conclusions such as "heavy-weight objects falls faster than light-weight objects." Based on intuition rather than proper dynamic analysis, it is not surprising that the explanation offered here is wrong. First, "*since only the 'downward' movement of the mass along the z axis transfers mechanical energy toward the tendons*"? No! Mechanical energy is transferred whenever there is a relative motion between the motor and the attached object. It seems that the authors mistook a vibration motor as a milling cutter, which "scrapes" the object when the mass tip approaches the surface. But in fact, the interaction of force takes place in every instant of the rotation. As I mentioned last time, the induced vibration takes place in both the x-axis and z-axis; in other words, the motor oscillates not only up and down but also left and right, continuously "pulling" the attached object

in every direction.

See the free body diagram below:

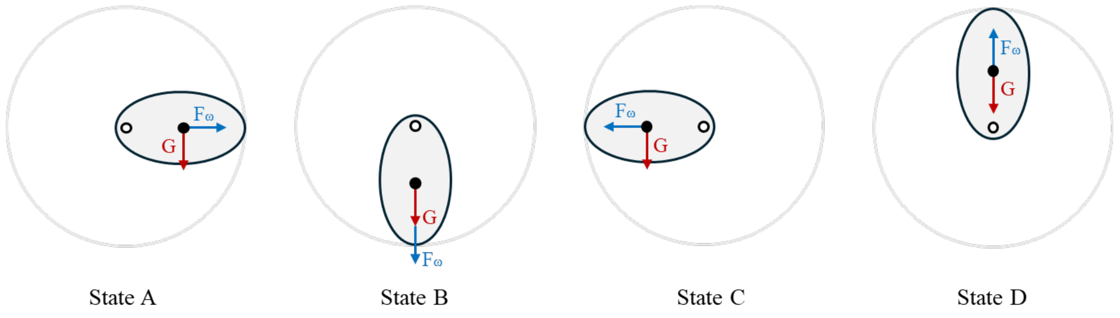

The vectors denote the forces applied to the attached object (e.g., the skin and tendons) as a result of the rotating mass: the gravitational force (G=mg) always points down, and the centrifugal force ($F_\omega = m\omega r^2$) always points away from the rotation center. Here, notice that the direction of rotation is not drawn, because it is irrelevant to the direction of the centrifugal force. Again, this is basic physics, I shouldn't need to explain this, but just in case, the mass pulls the object via the centrifugal force:

- rightward and downward (in any state between A and B),
- leftward and downward (in any state between B and C),
- leftward and upward (in any state between C and D),
- rightward and upward (in any state between D and A).

Based on the explanation and Fig 2, the authors misunderstood the process of vibration, believing that: for CW rotation, force is only applied to the object when the mass moves from State A to B; whereas for CCW rotation, force is only applied when the mass moves from State C to B. This is where the intuition went wrong.

Theoretical Explanation

The concept of shaft orbit is very nice, as it reflects the position of the shaft based on actual (not just theoretical) forces applied to the shaft, which is more complicated than my provided free body diagram. However, the authors misunderstood Fig 4 (which is Fig 10 in Ref 1), thinking that the two orbits are the results of different rotations. If you read Ref 1 carefully, it says "Figure 10 shows two orbits of the same size and shape, with different orientations and precessions" (suggesting the orbits themselves are of different orientations and precessions) — the arrowhead along the orbit indicates its own precession not the direction of rotation that induced this orbit. The point there is, if the precession is CCW and it was measured when the rotation was CCW, this orbit can be considered as a normal orbit (termed "forward precession"). But if the CCW precession was measured when the rotation was CW, then this orbit is abnormal (termed "reverse

precession”).

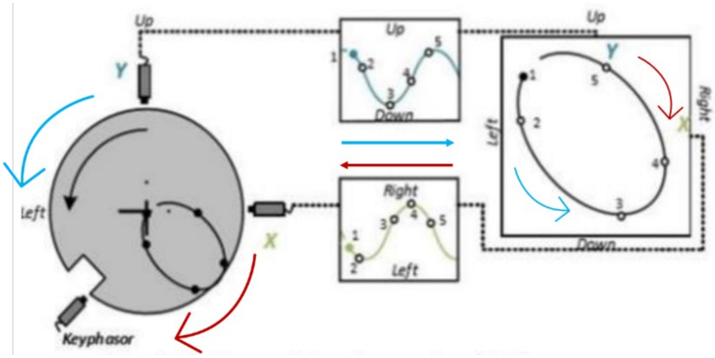

The key here is to distinguish between the precession of the orbit and the actual rotation of the mass. Just look at your Fig 3: the mass is rotating CCW, and the shaft produces the positions in the order of 1→2→3→4→5 in this order, leading to the orbit on the right side—in this case, the precession is CCW, going as 1→2→3→4→5. Now what if the mass rotates CW? Since the force will not change, the shaft would simply produce the positions reversely, going from 1→5→4→3→2. This will not change the size/shape/orientation of the orbit on the right side, but rather, its precession is changed to CW, going as 1→5→4→3→2.

Summing up, the direction of mass rotation affects certainly the direction of orbit precession, but not necessarily the direction of orbit orientation (at least not supported by Refs 1 and 2). I will not go on explaining how the direction of orbit precession is irrelevant to the induced vibration, but the authors should modify the text accordingly.

Experimental Evidence

I appreciate the authors for making efforts to provide experimental evidence. Indeed, it seems as if the orbit orientation is different between the mass rotation of CW and CCW, which is very surprising. I cannot think of a good explanation for this, but please make sure the sign of the readings is irrelevant to the rotational direction (if your accelerometer is powered by the motor circuits). Depending on how an accelerometer circuit is designed, the output sign might flip if the voltage is reversed. Simply knock on the motor when it rotates CW and CCW, and see if the induced peak is in the same direction.

As I mentioned last time it is appropriate to consider rotational direction as a factor as long as you demonstrate the difference with measurements. I do not know why this should be different theoretically, but the underlying reason is irrelevant to this work. The authors may present all results as they are, but please do not interpret this difference using the wrong intuition and theory: for example, “Shifting between clockwise and counterclockwise rotations likely changed the way the motor ‘hit’ underlying structures, as supported by orbital analyses of eccentric rotating mass systems [21, 22].”
