# Supplementary material for: Kinesthetic illusions induced by muscle tendon vibration: The orientation of the vibration motor as a new methodological factor?
Source: PLoS One. 2025 Jun 9;20(6):e0325737. doi: 10.1371/journal.pone.0325737 (PMC12148079; doi:10.1371/journal.pone.0325737)
Supplement: Attachment — Submitted filename: Response to Reviewers.docx [file pone.0325737.s004.docx]

**Point by point reply to Reviewer’s comments**

Dear Dr. Beaulieu,

Thank you for submitting your manuscript to PLOS ONE. After careful consideration, we feel that it has merit but does not fully meet PLOS ONE’s publication criteria as it currently stands. Therefore, we invite you to submit a revised version of the manuscript that addresses the points raised during the review process.

Please submit your revised manuscript by Oct 03 2024 11:59PM. If you will need more time than this to complete your revisions, please reply to this message or contact the journal office at [plosone@plos.org](mailto:plosone@plos.org). When you're ready to submit your revision, log on to <https://www.editorialmanager.com/pone/> and select the 'Submissions Needing Revision' folder to locate your manuscript file.

Please include the following items when submitting your revised manuscript:

- A rebuttal letter that responds to each point raised by the academic editor and reviewer(s). You should upload this letter as a separate file labeled 'Response to Reviewers'.
- A marked-up copy of your manuscript that highlights changes made to the original version. You should upload this as a separate file labeled 'Revised Manuscript with Track Changes'.
- An unmarked version of your revised paper without tracked changes. You should upload this as a separate file labeled 'Manuscript'.

If you would like to make changes to your financial disclosure, please include your updated statement in your cover letter. Guidelines for resubmitting your figure files are available below the reviewer comments at the end of this letter.

If applicable, we recommend that you deposit your laboratory protocols in protocols.io to enhance the reproducibility of your results. Protocols.io assigns your protocol its own identifier (DOI) so that it can be cited independently in the future. For instructions see: [https://journals.plos.org/plosone/s/submission-guidelines#loc-laboratory-protocols](https://can01.safelinks.protection.outlook.com/?url=https%3A%2F%2Fc05y1x9s.r.us-east-2.awstrack.me%2FL0%2Fhttps%3A%252F%252Fjournals.plos.org%252Fplosone%252Fs%252Fsubmission-guidelines%2523loc-laboratory-protocols%2F1%2F010f01916a03747c-5bea9e73-f20e-4587-9baa-9006a56ab3a7-000000%2FDhFmU854r5_NvP2ae-wDfT2-0-o%3D172&data=05%7C02%7Cllauzier%40etu.uqac.ca%7Cdb728af3f0a944b0238608dcc0386377%7Cc97978b1bd4c44b59bbb20215efdf611%7C0%7C0%7C638596595639956091%7CUnknown%7CTWFpbGZsb3d8eyJWIjoiMC4wLjAwMDAiLCJQIjoiV2luMzIiLCJBTiI6Ik1haWwiLCJXVCI6Mn0%3D%7C0%7C%7C%7C&sdata=vfYgJQUMF0LqhkChIeKJtL2pz6K8v5sPURc6gLQO2KE%3D&reserved=0). Additionally, PLOS ONE offers an option for publishing peer-reviewed Lab Protocol articles, which describe protocols hosted on protocols.io. Read more information on sharing protocols at [https://plos.org/protocols?utm_medium=editorial-email&utm_source=authorletters&utm_campaign=protocols](https://can01.safelinks.protection.outlook.com/?url=https%3A%2F%2Fc05y1x9s.r.us-east-2.awstrack.me%2FL0%2Fhttps%3A%252F%252Fplos.org%252Fprotocols%253Futm_medium%3Deditorial-email%2526utm_source%3Dauthorletters%2526utm_campaign%3Dprotocols%2F1%2F010f01916a03747c-5bea9e73-f20e-4587-9baa-9006a56ab3a7-000000%2FHwvFdkzyYQx1B1W36DjV3H_jHmE%3D172&data=05%7C02%7Cllauzier%40etu.uqac.ca%7Cdb728af3f0a944b0238608dcc0386377%7Cc97978b1bd4c44b59bbb20215efdf611%7C0%7C0%7C638596595639970866%7CUnknown%7CTWFpbGZsb3d8eyJWIjoiMC4wLjAwMDAiLCJQIjoiV2luMzIiLCJBTiI6Ik1haWwiLCJXVCI6Mn0%3D%7C0%7C%7C%7C&sdata=%2BXonLZn59et%2BuuXbsu2d50rXHNRV0FqRiMj6O2NOPjQ%3D&reserved=0).

Journal Requirements:

When submitting your revision, we need you to address these additional requirements.

1. Please ensure that your manuscript meets PLOS ONE's style requirements, including those for file naming. The PLOS ONE style templates can be found at 
<https://journals.plos.org/plosone/s/file?id=wjVg/PLOSOne_formatting_sample_main_body.pdf> and 
<https://journals.plos.org/plosone/s/file?id=ba62/PLOSOne_formatting_sample_title_authors_affiliations.pdf>

2. Please note that PLOS ONE has specific guidelines on code sharing for submissions in which author-generated code underpins the findings in the manuscript. In these cases, all author-generated code must be made available without restrictions upon publication of the work. Please review our guidelines at <https://journals.plos.org/plosone/s/materials-and-software-sharing#loc-sharing-code> and ensure that your code is shared in a way that follows best practice and facilitates reproducibility and reuse.

3. Thank you for stating the following financial disclosure: 
"This study was support by Ordre Professionnel de la Physiothérapie du Québec (to L. Lauzier : <https://oppq.qc.ca>), Réseau Intersectoriel en Santé de l’Université du Québec (to J. Abboud, F. Nougarou and L-D. Beaulieu : <https://risuq.uquebec.ca>), FRQ Fonds de Recherche du Québec – Santé (FRQS) under Grand number 297854 (to L-D. Beaulieu : <https://frq.gouv.qc.ca/sante/>) and Réseau Provincial de Recherche en Adaptation-Réadapation (REPAR) (to L. Lauzier : <https://repar.ca>)."

Please state what role the funders took in the study.  If the funders had no role, please state: ""The funders had no role in study design, data collection and analysis, decision to publish, or preparation of the manuscript."" 
If this statement is not correct you must amend it as needed. 
Please include this amended Role of Funder statement in your cover letter; we will change the online submission form on your behalf.

4. We note that your Data Availability Statement is currently as follows: All relevant data are within the manuscript and its Supporting Information files.

Please confirm at this time whether or not your submission contains all raw data required to replicate the results of your study. Authors must share the “minimal data set” for their submission. PLOS defines the minimal data set to consist of the data required to replicate all study findings reported in the article, as well as related metadata and methods (<https://journals.plos.org/plosone/s/data-availability#loc-minimal-data-set-definition>).

For example, authors should submit the following data:

- The values behind the means, standard deviations and other measures reported;
- The values used to build graphs;
- The points extracted from images for analysis.

Authors do not need to submit their entire data set if only a portion of the data was used in the reported study.

If your submission does not contain these data, please either upload them as Supporting Information files or deposit them to a stable, public repository and provide us with the relevant URLs, DOIs, or accession numbers. For a list of recommended repositories, please see <https://journals.plos.org/plosone/s/recommended-repositories>.

If there are ethical or legal restrictions on sharing a de-identified data set, please explain them in detail (e.g., data contain potentially sensitive information, data are owned by a third-party organization, etc.) and who has imposed them (e.g., an ethics committee). Please also provide contact information for a data access committee, ethics committee, or other institutional body to which data requests may be sent. If data are owned by a third party, please indicate how others may request data access.

5. Please include your full ethics statement in the ‘Methods’ section of your manuscript file. In your statement, please include the full name of the IRB or ethics committee who approved or waived your study, as well as whether or not you obtained informed written or verbal consent. If consent was waived for your study, please include this information in your statement as well.

6. Please include a separate caption for each figure in your manuscript.

7. Please include your tables as part of your main manuscript and remove the individual files. Please note that supplementary tables (should remain/ be uploaded) as separate ""supporting information"" files

**Reviewers' comments: Reviewer's Responses to Questions**

**Comments to the Author**

1. Is the manuscript technically sound, and do the data support the conclusions?

The manuscript must describe a technically sound piece of scientific research with data that supports the conclusions. Experiments must have been conducted rigorously, with appropriate controls, replication, and sample sizes. The conclusions must be drawn appropriately based on the data presented.

Reviewer #1: Partly

Reviewer #2: Yes

2. Has the statistical analysis been performed appropriately and rigorously?

Reviewer #1: I Don't Know

Reviewer #2: I Don't Know

3. Have the authors made all data underlying the findings in their manuscript fully available?

The [PLOS Data policy](https://can01.safelinks.protection.outlook.com/?url=http%3A%2F%2Fc05y1x9s.r.us-east-2.awstrack.me%2FL0%2Fhttp%3A%252F%252Fwww.plosone.org%252Fstatic%252Fpolicies.action%2523sharing%2F1%2F010f01916a03747c-5bea9e73-f20e-4587-9baa-9006a56ab3a7-000000%2FV_RVl8qrGIvGSuLXILQmm3EaAqc%3D172&data=05%7C02%7Cllauzier%40etu.uqac.ca%7Cdb728af3f0a944b0238608dcc0386377%7Cc97978b1bd4c44b59bbb20215efdf611%7C0%7C0%7C638596595639982420%7CUnknown%7CTWFpbGZsb3d8eyJWIjoiMC4wLjAwMDAiLCJQIjoiV2luMzIiLCJBTiI6Ik1haWwiLCJXVCI6Mn0%3D%7C0%7C%7C%7C&sdata=gDwlM%2BFBDHqi63nc0sdWG5pZrYsnVawAvph91OnBxO4%3D&reserved=0) requires authors to make all data underlying the findings described in their manuscript fully available without restriction, with rare exception (please refer to the Data Availability Statement in the manuscript PDF file). The data should be provided as part of the manuscript or its supporting information, or deposited to a public repository. For example, in addition to summary statistics, the data points behind means, medians and variance measures should be available. If there are restrictions on publicly sharing data—e.g. participant privacy or use of data from a third party—those must be specified.

Reviewer #1: No

Reviewer #2: Yes

4. Is the manuscript presented in an intelligible fashion and written in standard English?

PLOS ONE does not copyedit accepted manuscripts, so the language in submitted articles must be clear, correct, and unambiguous. Any typographical or grammatical errors should be corrected at revision, so please note any specific errors here.

Reviewer #1: Yes

Reviewer #2: Yes

5. Review Comments to the Author

Please use the space provided to explain your answers to the questions above. You may also include additional comments for the author, including concerns about dual publication, research ethics, or publication ethics. (Please upload your review as an attachment if it exceeds 20,000 characters)

**Reviewer #1:**

***Comment #1****:* It is interesting study to investigate the effect of rotational direction of vibration motor on kinesthetic illusion. However, necessary details are missing for readers to understand the result. First, it is hard to grasp the experimental procedure. Please add a proper figure describing the experimental procedure. Note that experimental procedure is critical for the kinesthetic illusion test, as the previous posture/movement of the upper limb critically affects the illusion of wrist joint.

**Answer:** We thank the Reviewer for their comments and suggestions to improve the understanding of our article. A new figure was produced to illustrate the overall experimental procedure *(see figure 2. Experimental procedure).* We also recognize that the limb position is an important factor that can affect kinesthetic illusion. We added some details in the manuscript:

- Lines (118-125): “The participant was seated in a standard chair with their forearm supported on a table (shoulder and elbow slightly flexed about 45 degrees). The wrist was supported by the experimenter to control its angular position and to ensure an optimal relaxation throughout the experiment. An elastic strap was installed at the level of the metacarpal heads to reduce tactile feedback from the experimenter holding the hand. A small flexible goniometer was taped on the skin, secured under the elastic strap and aligned with standard anatomic landmarks (i.e. parallel with radius bone and with second metacarpal bone) to keep track of the wrist angular position (fig. 1).”

***Comment #2:*** Second, it is hard to understand the results because of the unfamiliar evaluation method (SKIP), which has not been well explained in the manuscript.
Although the SKIP has been introduced in authors' prior publication, authors should assume that most of the readers would not be familiar to that evaluation method. Please add a figure describing the method (what each score means?). Also, please note that direction and amplitude should be clearly investigated, to describe the full aspects of the kinesthetic illusion. For example, if the illusion is "perfectly clear and precise", what that means in terms of angular illusion (how much differently the wrist angle was perceived)? If authors can provide any clue matching the strength of illusion and the angular illusion, that would be helpful for the readers to understand the impact of the intervention (vibration). With these two information (exp procedure and exp result) well provided, I can fully review the manuscript.

**Answer:** We agree that readers can be unfamiliar with SKIP which has been only recently proposed (2020) [1]. Of note, SKIP was based on best practices and known factors of influence from renown publications in the field of VIB-induced kinesthetic illusions. All details on how the SKIP procedure was created based on the literature can be found in the 2020 paper. We chose to add details in the manuscript instead of creating another figure since we already have 3 figures. For the present manuscript, a section dedicated to SKIP explications was added in *Materials and Methods*:

- Lines (134-156): Prior to the experiment, a few VIB trials were tested with eyes open to ensure setup comfort and habituation to VIB cutaneous sensation. Each VIB trial used the following parameters: 80 Hz frequency, 1 mm amplitude and 10 s duration [2, 3]. Standardized instructions were given to the participant: “*The test is designed to evaluate how well you can perceive movements at the joint during the vibration. Beyond the sensation of vibration, you will have to tell whether a movement occurred or not and if yes, to give specific details on the perceived movement.”*. The first step of SKIP is the determination of an optimal joint position to perceive the clearest illusion. VIB is first applied at an arbitrary sufficient angle to slightly stretch the targeted tendon. The evaluator then tests different wrist joint angles in the sagittal plane (flexion/extension) using five degrees increments. The angle at which the clearest illusions are perceived is noted by the experimenter using the goniometer strapped on the wrist and used throughout the rest of the experiment. The next step is to assess the illusion clearness/precision and direction at the optimal joint position. Three consecutive trials are realized and for each trial, the subject rate the clearness/precision (perfectly clear and precise = 3; moderately clear/precise = 2; vague and not precise = 1; no illusion = 0) and direction (illusion in the expected direction (i.e. which would stretch the vibrated tendons) = 1; any other direction = 0). In addition, a VAS is used to evaluate the perceived speed/amplitude of the illusions, where scores near 0 relates to a very low speed/amplitude and scores toward 10 would refer to a very high speed/amplitude [4]. In addition to rate the direction score using the SKIP grading system, the evaluator asked participants to qualitatively describe the perceived direction and/or reproducing what they felt with their wrist. This information was used to determine the frequency of occurrence for the different illusion directions.

To answer the second part of the Reviewer’s comment, illusion clearness, direction and speed/amplitude were measured separately as they represent different characteristics that could vary independently to each other. For example, participants can perceive an illusion as "perfectly clear and precise" even if the angular illusory movement is small. The perception of a kinesthetic illusion is a complex experience and we agree with the Reviewer that our work should more clearly address how the different characteristics of the illusions (direction, amplitude, etc) relate (or not) to each other. After a careful revision of our data, we found an error in the pooled data for the Lateromedial orientation in Table 2. Indeed, the previous version of Table 2 was showing, for the dominant and non-dominant sides, 25^th^-75^th^ quartiles ranging between 1 and 3 points on the 3-points scale of clearness, and a median at 2 points. We confirm that these numbers were correct after a careful verification. However, the pooled data on Table 2 showed a median at 1 pt and 25^th^-75^th^ range from 0-2 pts, which makes no sense considering that data are pooled from the dominant and non-dominant sides. We re-calculated and the actual median is 2 pts with an 25^th^-75^th^ interquartile range of 2-3 pts. We took time to double check all data (now provided in a Supplement file) and results from our experiment and found no other error. We sincerely apologize for this mistake. Fortunately, the Reviewer’s comment helped us realize the error and make the required corrections which can be found (marked in red) in the new Table 2 in the manuscript lines/pages 213-216.

Based on these corrected data, results demonstrated that dominant, non-dominant and pooled clearness scores were similar between all 4 conditions of VIB orientations, which also agrees with the findings for speed/amplitude. Conversely, the lateromedial orientation was shown as the one leading to more unexpected directions compared to the distoproximal and proximodistal (Table 2). Furthermore, the lateromedial orientation showed a higher variability of directions perceived as described qualitatively by the participants (Table 3). So, our work does not really underscore that illusions perceived using the lateromedial rotation are less clear, slower or of less amplitude, but rather that they more often lead to unexpected and variable directions. This nuance is important, since if the lateromedial orientation led to vague and weak illusions, then one could have question if their higher variability of direction only mirrors a lesser capacity of the participant to clearly identify the perceived direction of illusion. Our results do not agree with this explanation, since illusions using the lateromedial orientation were of similar clearness and speed/amplitude than the other orientations. The following additions were made in the *Discussion* section about this topic:

- Lines (289-295): Interestingly, the higher variability of directions with the lateromedial orientation is probably not caused by more vague and weaker illusions that could have challenged the ability of the participants to identify the precise direction. Indeed, illusions were of similar clearness and speed/amplitude between all four orientations. This further supports that the lateromedial orientation is effective in vibrating tendons and creating kinesthetic illusions, but that this particular orientation preferentially stimulates different tendons (like the flexor carpi radialis) compared to the other orientations.

**Reviewer #2:** The article is clear, well-structured, and thoughtfully organized, making it accessible to both specialists and non-specialists in the field. The authors have successfully presented their research in a logical sequence, ensuring that each section builds upon the previous one. Their adherence to both research and publication ethics is evident throughout the paper, as they have demonstrated transparency in their methodology and respect for ethical guidelines, particularly in data handling and participant confidentiality. The study's findings contribute meaningfully to the existing body of knowledge, offering new insights and potential directions for future research. Given the article’s clarity, ethical rigor, and valuable contribution, I find no areas that require revision or improvement and have no further comments.

**Answer:** We thank the Reviewer for their time and positive comments regarding our article.

6. PLOS authors have the option to publish the peer review history of their article ([what does this mean?](https://can01.safelinks.protection.outlook.com/?url=https%3A%2F%2Fc05y1x9s.r.us-east-2.awstrack.me%2FL0%2Fhttps%3A%252F%252Fjournals.plos.org%252Fplosone%252Fs%252Feditorial-and-peer-review-process%2523loc-peer-review-history%2F1%2F010f01916a03747c-5bea9e73-f20e-4587-9baa-9006a56ab3a7-000000%2FQVbX147axtYEooP40DoL3wMsCO8%3D172&data=05%7C02%7Cllauzier%40etu.uqac.ca%7Cdb728af3f0a944b0238608dcc0386377%7Cc97978b1bd4c44b59bbb20215efdf611%7C0%7C0%7C638596595639992896%7CUnknown%7CTWFpbGZsb3d8eyJWIjoiMC4wLjAwMDAiLCJQIjoiV2luMzIiLCJBTiI6Ik1haWwiLCJXVCI6Mn0%3D%7C0%7C%7C%7C&sdata=joCX5SzSIOsWDFoHY0Otb4Td0BWWnD%2BJveLEHxeGSIU%3D&reserved=0)). If published, this will include your full peer review and any attached files.


If you choose “no”, your identity will remain anonymous but your review may still be made public.

**Do you want your identity to be public for this peer review?** For information about this choice, including consent withdrawal, please see our [Privacy Policy](https://can01.safelinks.protection.outlook.com/?url=https%3A%2F%2Fc05y1x9s.r.us-east-2.awstrack.me%2FL0%2Fhttps%3A%252F%252Fwww.plos.org%252Fprivacy-policy%2F1%2F010f01916a03747c-5bea9e73-f20e-4587-9baa-9006a56ab3a7-000000%2Fx_Og6ZJg7eK8EZdv0fRCXlgUZAg%3D172&data=05%7C02%7Cllauzier%40etu.uqac.ca%7Cdb728af3f0a944b0238608dcc0386377%7Cc97978b1bd4c44b59bbb20215efdf611%7C0%7C0%7C638596595640002856%7CUnknown%7CTWFpbGZsb3d8eyJWIjoiMC4wLjAwMDAiLCJQIjoiV2luMzIiLCJBTiI6Ik1haWwiLCJXVCI6Mn0%3D%7C0%7C%7C%7C&sdata=Z7tx7UXkJr1g3nduM6ZT2%2FR86RVZU2VDt2CCYPJY1dY%3D&reserved=0).

Reviewer #1: **Yes:** Hangue Park

Reviewer #2: No

**References**

1.	Beaulieu, L.D., et al., *A new method to elicit and measure movement illusions in stroke by means of muscle tendon vibration: the Standardized Kinesthetic Illusion Procedure (SKIP).* Somatosens Mot Res, 2020. **37**(1): p. 28-36.

2.	Roll, J.P.D. and J.P. Vedel, *Kinaesthetic role of muscle afferents in man, studied by tendon vibration and microneurography.* Experimental Brain Research, 1982. **47**(2): p. 177-190.

3.	Calvin-Figuiere, S., et al., *Antagonist motor responses correlate with kinesthetic illusions induced by tendon vibration.* Exp Brain Res, 1999. **124**(3): p. 342-50.

4.	Scott, J. and E.C. Huskisson, *Vertical or horizontal visual analogue scales.* Ann Rheum Dis, 1979. **38**(6): p. 560.
