# Supplementary material for: Kinesthetic illusions induced by muscle tendon vibration: The orientation of the vibration motor as a new methodological factor?
Source: PLoS One. 2025 Jun 9;20(6):e0325737. doi: 10.1371/journal.pone.0325737 (PMC12148079; doi:10.1371/journal.pone.0325737)
Supplement: Attachment — Submitted filename: Response to reviewers_final.docx [file pone.0325737.s006.docx]

**Point by point reply**

**Comments from the Editor**

Dear Dr. Beaulieu,

Thank you for submitting your manuscript to PLOS ONE. After careful consideration, we feel that it has merit but does not fully meet PLOS ONE’s publication criteria as it currently stands. Therefore, we invite you to submit a revised version of the manuscript that addresses the points raised during the review process.

Please submit your revised manuscript by Feb 13 2025 11:59PM. If you will need more time than this to complete your revisions, please reply to this message or contact the journal office at [plosone@plos.org](mailto:plosone@plos.org). When you're ready to submit your revision, log on to <https://www.editorialmanager.com/pone/> and select the 'Submissions Needing Revision' folder to locate your manuscript file.

Please include the following items when submitting your revised manuscript:

- A rebuttal letter that responds to each point raised by the academic editor and reviewer(s). You should upload this letter as a separate file labeled 'Response to Reviewers'.
- A marked-up copy of your manuscript that highlights changes made to the original version. You should upload this as a separate file labeled 'Revised Manuscript with Track Changes'.
- An unmarked version of your revised paper without tracked changes. You should upload this as a separate file labeled 'Manuscript'.

If you would like to make changes to your financial disclosure, please include your updated statement in your cover letter. Guidelines for resubmitting your figure files are available below the reviewer comments at the end of this letter.

If applicable, we recommend that you deposit your laboratory protocols in protocols.io to enhance the reproducibility of your results. Protocols.io assigns your protocol its own identifier (DOI) so that it can be cited independently in the future. For instructions see: [https://journals.plos.org/plosone/s/submission-guidelines#loc-laboratory-protocols](https://can01.safelinks.protection.outlook.com/?url=https%3A%2F%2Ftrack.editorialmanager.com%2FCL0%2Fhttps%3A%252F%252Fjournals.plos.org%252Fplosone%252Fs%252Fsubmission-guidelines%2523loc-laboratory-protocols%2F1%2F010f01941998874c-895bd655-1518-4664-8914-c73fdf223837-000000%2FOPBJAJL6SIjr-aamVawqWyZYJpd_3oxYihnlY5fmOPM%3D191&data=05%7C02%7Cllauzier%40etu.uqac.ca%7C394b1974303947475fbf08dd2e68776d%7Cc97978b1bd4c44b59bbb20215efdf611%7C0%7C0%7C638717748710314507%7CUnknown%7CTWFpbGZsb3d8eyJFbXB0eU1hcGkiOnRydWUsIlYiOiIwLjAuMDAwMCIsIlAiOiJXaW4zMiIsIkFOIjoiTWFpbCIsIldUIjoyfQ%3D%3D%7C0%7C%7C%7C&sdata=ipa3LNj6kqCgBbblQvAsNNL%2BMkOFYt0Y%2F6IiUjG08lQ%3D&reserved=0). Additionally, PLOS ONE offers an option for publishing peer-reviewed Lab Protocol articles, which describe protocols hosted on protocols.io. Read more information on sharing protocols at [https://plos.org/protocols?utm_medium=editorial-email&utm_source=authorletters&utm_campaign=protocols](https://can01.safelinks.protection.outlook.com/?url=https%3A%2F%2Ftrack.editorialmanager.com%2FCL0%2Fhttps%3A%252F%252Fplos.org%252Fprotocols%253Futm_medium%3Deditorial-email%2526utm_source%3Dauthorletters%2526utm_campaign%3Dprotocols%2F1%2F010f01941998874c-895bd655-1518-4664-8914-c73fdf223837-000000%2FnmPrKS3-k6HGDb13qAyC0eAD12vpiSxRQO5KQ-jrZEY%3D191&data=05%7C02%7Cllauzier%40etu.uqac.ca%7C394b1974303947475fbf08dd2e68776d%7Cc97978b1bd4c44b59bbb20215efdf611%7C0%7C0%7C638717748710335073%7CUnknown%7CTWFpbGZsb3d8eyJFbXB0eU1hcGkiOnRydWUsIlYiOiIwLjAuMDAwMCIsIlAiOiJXaW4zMiIsIkFOIjoiTWFpbCIsIldUIjoyfQ%3D%3D%7C0%7C%7C%7C&sdata=brtjkEcA12PIxs3YRU9zvoWtjCjWcmypJEjmKk1k2Qg%3D&reserved=0).

We look forward to receiving your revised manuscript.

Kind regards,

Monika Błaszczyszyn
Academic Editor
PLOS ONE

Journal Requirements:

Please review your reference list to ensure that it is complete and correct. If you have cited papers that have been retracted, please include the rationale for doing so in the manuscript text, or remove these references and replace them with relevant current references. Any changes to the reference list should be mentioned in the rebuttal letter that accompanies your revised manuscript. If you need to cite a retracted article, indicate the article’s retracted status in the References list and also include a citation and full reference for the retraction notice.

[Note: HTML markup is below. Please do not edit.]

- **Answer:** We thank the Editor for letting us revise and improve further our manuscript. We have strictly followed the Journal Requirements and hope that the Editors and Reviewers find the revised version suitable for publication in PLOS ONE.

**Comments from Reviewer #2**

Comment #1: The article is clear, well-structured, and thoughtfully organized, making it accessible to both specialists and non-specialists in the field. The authors have successfully presented their research in a logical sequence, ensuring that each section builds upon the previous one. Their adherence to both research and publication ethics is evident throughout the paper, as they have demonstrated transparency in their methodology and respect for ethical guidelines, particularly in data handling and participant confidentiality. The study's findings contribute meaningfully to the existing body of knowledge, offering new insights and potential directions for future research. Given the article’s clarity, ethical rigor, and valuable contribution, I find no areas that require revision or improvement and have no further comments.

- **Answer:** We thank the Reviewer for their positive comments on the revised manuscript and for their time reviewing our work.

**Comments from Reviewer #3**

Comment #1: Although this manuscript generally satisfies the seven criteria of PLOS ONE publication, there are concerns regarding important aspects of the study. This work focuses on how the orientation of the vibration motor influences kinesthetic illusion, which could be somewhat useful to certain researchers and clinicians. However, before going through the Methods section, there is an immediate question coming to mind: Does flipping a motor’s orientation affect the induced vibration? As shown in the figure below (https://www.precisionmicrodrives.com/ab-014), when an eccentric rotating mass (ERM) vibration motor is placed on the skin, the induced vibration can be regarded as “bidirectional”: one perpendicular to the skin (z-axis), and one perpendicular to the motor rotation axis while parallel to the skin (x-axis); no vibration is induced along the motor rotation axis (y axis). Most importantly, the orientation of the y- axis is irrelevant to the induced vibration: If the y-axis is rotated around the z-axis by 180°, x-axis is indeed flipped, but since the motor oscillates back and forth along the x-axis, vibration is unaffected. Similarly, vibration is independent of the direction of mass rotation (CW or CCW).

Fig 1.

That said, a fundamental issue in the study design is that the induced vibrations in Figs 3a (proximodistal) and 3b (distoproximal) are essentially the same (so are Figs 3c and 3d), and the study is in fact comparing two types of vibrations (y-axis rotated around z-axis by 0° and 90°) instead of four (0°, 90°, 180°, 270°). While I agree flipping motor orientation should be controlled, this work treats it as a key variable without justification. The mechanism of the ERM vibration motor should be better explained, and the authors will need to demonstrate how and why flipping motor orientation could have affected the induced/perceived vibration, either by citation or measurement. Only then it is appropriate to investigate the impact on kinesthetic illusion.

- **Answer:** We thank the Reviewer for their insightful comment, which calls for a clearer and more detailed explanation of the study’s rationale and interpretation of the results. The description provided by the Reviewer about eccentric rotating mass (ERM) motors, along with Fig 1 illustrating the vibration axes, is accurate. In fact, this information originates from our ERM’s manufacturer which provided a series of technical posts to describe how an ERM works <https://www.precisionmicrodrives.com/technical-resources>.

Further, it is true that we only turned the motor around itself (y-axis rotated around z-axis) between 0 and 90 deg. However, the Reviewer’s statement that “the vibration of an ERM motor is independent of the direction of mass rotation (clockwise, CW or counterclockwise CCW)” is incorrect. Although we agree that it has never been properly addressed in the field of muscle tendon vibration (hence the importance of our work), it was demonstrated by publications and technical reports from industrial engineering (described below). In the next paragraphs, we will argument intuitively, theoretically, and experimentally that the rotational direction of the ERM (CW and CCW) does influence the effects of muscle tendon vibration, hence supporting that our study did test four conditions of orientation and not just two. We will also describe all changes made to the manuscript in response to the Reviewer’s comment to further clarify this important aspect.

**Intuitive Explanation**

As mentioned on the manufacturer’s website, the centripetal force of the unbalanced mass in an ERM is asymmetrical, resulting in a net centrifugal force that causes the motor to move. Since the motor rotates, it is continuously displaced by these asymmetrical forces, thereby generating vibration. Although an ERM does indeed oscillate in two planes (x-axis and z-axis according to Fig. 1), these oscillations are not independent from one another – there are linked to the rotational movement of the mass. So, if we measure the real-time movement of the vibrator it would always have an instantaneous ‘x’ and ‘z’ direction that change from point to point along the circular motion of the mass. Since only the ‘downward’ movement of the mass along the z axis transfers mechanical energy toward the tendons, then the ‘stimulating’ phase of the circular motion of the mass happens either when the mass is located to the right or to the left of the motor’s center depending on the rotational direction of the mass (CW or CCW). With this in mind, let us consider our motor, shown in Fig. 2. When the mass rotates CCW, the centrifugal force and location of the mass when it moves toward the tendon create a slight imbalance towards the left, accentuating mechanical energy transfer to the left of the motor (Fig. 2a). Similarly, when the mass rotates clockwise (CW), the centrifugal force and location of the mass cause a slight imbalance towards the right, which in turn accentuates the vibrations to the right of the motor (Fig. 2b). The change in centrifugal force direction is directly linked to the change in rotational direction of the unbalanced mass, much like the sensation of being pushed outward when turning left or right in a moving vehicle. For the sake of simplification let us call this accentuating effect the main ‘angle of attack’ of the vibration.

Fig. 2. Change in the vibration angle of attack depending on the rotation direction due to the change in the direction of the centrifugal force. This illustration was made from a photo of our vibration device.

Considering this, it is incorrect to assume that a rotating motor produces identical vibrations regardless of the rotational direction of the mass. Further evidence of the impact of changing between CW and CCW is described in mathematical models and technical reports from engineering studies, as described below.

**Theoretical Explanation**

A more theoretical way to demonstrate that vibratory behaviour varies with the rotational direction of the eccentric mass in an ERM motor is to calculate and observe the motor’s vibratory orbit when the motor rotates in both directions: CW and CCW. Orbit analysis is extensively used in industry to diagnose the vibratory behaviour of rotating machinery and has been studied in numerous works such as [1.1] and [1.2]. The orbit of any rotating motor, including ERMs, results from the combination of vibratory components along two axes, such as the X and Z axes illustrated by the Reviewer in Fig. 1. These two components can be defined as sinusoidal waves with an amplitude, frequency, and phase. By measuring and combining these two waves in real time during motor operation, a vibratory orbit can be constructed (see Fig. 3 – *note that the two axes are identified as X and Y in this Figure instead of X and Z*).

Fig. 3. Presentation of the orbit construction form [1.1].

This orbit reflects the overall vibratory signature generated by a rotating motor and its propagation to the surrounding structures. Indeed, the vibratory waves along the two axes should not be considered independently. The overall vibratory behaviour depends on both the amplitude of these waves and their phase shift, as the forces combine along X and Z. In [1.2], the author presents a method to calculate this orbit, explains how it provides insights into the motor’s condition, and illustrates how the orbit shape changes with the CW-CCW rotational direction (Fig. 4).

Fig. 4: Illustration of the orbit behavior of a running motor based on the change in rotation (CCW and CW) from [1.2].

Fig. 4 from this study supports the intuitive explanation described above, although not in the context of muscle tendon vibration. In CW rotation, the vibratory forces along X and Z, influenced by the centrifugal force, produce an elliptical vibratory orbit tilted to the right. In CCW rotation, these forces generate an elliptical orbit tilted to the left. In addition to the changing angle of attack, the orbit’s orientation also changes between CW and CCW directions. Thus, the vibration produced by a rotating motor such as the ERM has a different attack angle and an overall orbital shape that change with the motor’s rotational direction. While the intuitive and theoretical arguments presented above are supporting that CW and CCW rotational direction do present key differences, it remains unclear how they manifested in our vibration setup and influenced the effects of muscle tendon vibration. The next sections will further expand on these points and present experimental data measuring the orbit of our ERM system.

**Experimental Evidence**

As shown in Fig. 5, an accelerometer circuit was positioned on our motor. This circuit was initially added to provide accurate, real-time readings of vibration frequencies during stimulation. Additionally, this sensor allows us to calculate and observe our vibrating motor’s orbit in real time, providing deeper insights into its vibratory behaviour. Using acceleration measurements along the X and Z axes from the sensor, we extracted the vibratory waves from these two axes and combined them to reconstruct the orbit. Fig. 5 presents a schematic description of the orbit construction process.

Fig 5: Process of calculating the vibration orbit from our accelerometer-based system.

To support the claim that the vibratory behaviour of our ERM motor varies with the rotational direction, Fig. 6 presents the obtained orbit shapes in CW and CCW conditions. As shown in these figures, based directly on experimental data collected over one minute, the vibratory orbit of our motor exhibits different elliptical shapes depending on the rotational direction, consistent with both the intuitive and theoretical explanations.

Fig. 6: Example of obtained orbit shapes in CW and CCW conditions at a vibration frequency of 50 Hz, based on real experimental values from our VMT system.

**Effect on muscle tendon vibration**

We have now demonstrated that changing the rotational direction of the ERM motor induces a different vibratory behaviour, particularly in terms of its ‘angle of attack’ and orbital shape over the wrist. Because the vibratory behavior is different in the two rotation directions, our study did examine four motor position conditions (0° - CW, 0° - CCW, 90°- CW, 90°- CCW), and not just two as suggested by the Reviewer. Since the lateral edges of the plastic casing holding our motor are approximately 2 cm apart (the vibrator contacts the participant with a rectangular shape of 1.8 x 2.5 cm), this angle of attack shifts by about 2 cm depending on the rotational direction: either slightly more to the left or more to the right. As argued in our manuscript’s discussion (lines 327-383), the location of anatomical structures under the vibrator could explain why different perceptions were found between lateromedial (more pressure applied on the lateral edge) and mediolateral (more pressure on the medial edge).

Tendons of finger and wrist flexors are not localized uniformly; most of them are located between the midline and the medial (ulnar) sections of the wrist (cf. green line in Fig. 7 below). Further, compared to the flexor carpi ulnaris, the flexor carpi radialis is located slightly more toward the midline (cf. pink lines in Fig. 7) hence in more direct contact under the vibrator (cf. grey box in Fig. 7). This could explain why illusory perceptions of ulnar deviations (caused by vibrating the flexor carpi radialis) were more frequently reported with the lateromedial conditions as compared to illusions of radial deviations (caused by vibrating the flexor carpi ulnaris).

Midline

Fig 7. Cross-sectional anatomical representation of the wrist https://www.researchgate.net/publication/323863085/figure/fig1/AS:614222484500492@1523453460832/Human-wrist-cross-section-5.png

Thus, there are valid justifications supporting the study’s aim and rationale regarding the importance of better understanding and controlling the rotational direction of the mass in ERM vibrators. Some of the ideas presented here in response to the Reviewer’s comment were added to the manuscript to clarify the study’s rationale and interpretations based on the Results. These can be found in lines 78-102, 185-188; 299-383. Importantly, we stressed out in the Discussion that the effect of changing the rotational direction only applies to unbalanced ERM systems and not to other vibratory device that uses different methods of vibration restricted to an uniaxial direction (z-axis only; cf lines 370-371). We also further explored other potential factors of influence, such as the size and shape of the vibrator and the contribution of skin mechanoreceptors, as found in lines 312-321, 372-377.

References:

[1.1] Jordan, M. A. (2003). What Are Shaft Orbits Anyway? <https://pdfcoffee.com/shaft-orbits-5-pdf-free.html>

[1.2] Da Costa, C., Da Gama, R. S., Nascimento, C. E., Brandão, I. M., De Medeiros, E. C., & Mathias, M. H. (2018). Orbit Analysis For Imbalance Fault Detection In Rotating

Machinery. *IOSR Journal of Electrical and Electronics Engineering*, *13*(1), 43-53. <https://doi.org/10.9790/1676-1301044353>

Changes made to the manuscript relating to Comment #1:

**Introduction**

“However, information about how the vibration motor should be placed over the tendon is mostly lacking. Vibration motors come with different sizes, shapes, and mechanisms to create vibrations. Many of those commercially available are using a rotating system named eccentric rotating mass on which a small weight is mounted off-center from the rotational axis [20]. The centripetal force of the unbalanced mass is asymmetrical, resulting in a net centrifugal force that causes the motor to move, thereby generating vibration [20]. The combination of speed of rotation with the distance and weight of the eccentric mass overall results in the observed vibration frequency and amplitude [21]. For maximal energy transfer to the underlying tendon, the system must be placed so that the internal mass rotates in a way that makes the vibrator moves up and down perpendicularly to the skin. However, since the mass moves in a circular way, the vibrator also oscillates back and forth parallel to the skin and tendons [20]. The overall vibratory behavior can thus be represented as two sinusoidal waves with an amplitude, frequency, and phase that are propagating along the two axes (i.e. perpendicular and parallel to the tendon) [21, 22]. From mathematical models and real-time measurements of these two waves, a vibratory orbit can be constructed. Orbit analysis is extensively used in industry to diagnose abnormal vibratory behaviour of rotating machinery [21, 22], but has never been considered as a potential methodological factors to control in muscle tendon vibration. Yet, orbit analysis showed that although the eccentric mass rotates in a circular pattern, the asymmetrical forces and phase shift between the two waves rather produce an elliptical vibratory orbit [21, 22]. The orientation of the elliptical orbit changes depending on the rotational direction of the eccentric mass (that is, clockwise and counterclockwise) [21, 22]. In the case of muscle tendon vibration for example, the longer axis of the ellipse would be pointing down toward the tendons and slightly tilted to the right or to the left depending on the rotational direction of the eccentric mass.

Altogether, changing the vibration’s position and the rotational direction of the eccentric mass could change the location and direction of the forces transmitted to the targeted tendons. To date, no study verified whether the rotational direction and placement of the vibrator influence or not the perceived illusions - these methodological choices remain arbitrary. Considering the potential of vibration-induced illusions for diagnostic and therapeutic purposes, all potential influencing factors should be rigorously investigated to ensure that a low sensory perception or limited response to treatment are ascribed to meaningful clinical interpretations and not methodological issues.”

**Methods**

“SKIP measures were obtained for both the dominant and non-dominant hands in four conditions consisting of different rotational orientations of the vibrating motor, presented in a random order between participants (Fig 2). These directions were defined by how the rotating mass of the motor would hit the tendons first according to the standard anatomical position: (i) proximodistal, (ii) distoproximal, (iii) mediolateral and (iv) lateromedial (see Fig 3). Of note, the vibrator’s position was exactly the same for the proximodistal and distoproximal conditions, as it was the same for mediolateral and lateromedial conditions – the only difference was the clockwise and counterclockwise rotational direction of the eccentric mass.”

**Discussion**

“The high success rates and limited contrasts found between the four conditions underscore that even if the position and orientation of an eccentric rotating mass vibrator stays uncontrolled, a relatively high efficacy for inducing kinesthetic illusions can still be expected. However, if a specific and reproducible direction of illusion is desired, for example a wrist extension, then our study stresses out the importance of better controlling the vibrator’s position and rotational orientation. The lateromedial condition was less effective in producing the expected illusions of wrist extension, especially compared to the distoproximal orientation. Also, participants reported more frequently movements that included an ulnar component when using the lateromedial orientation. We propose technological and anatomical explanations for these findings. We fixed our vibrating motor in a 3D-printed plastic cover that fit the shape of the motor. This resulted in a surface of contact with the participant having a slight rectangular shape of 1.8 x 2.5 cm. So, flipping the vibrator’s orientation by 90 degrees between the distoproximal (or proximodistal) and the lateromedial (or mediolateral) conditions slightly changed the surface of contact over the tendons. The larger 2.5 cm side of the system when using distoproximal/proximodistal position could have better covered all tendons from wrist flexors compared to the smaller 1.8 cm side used for the lateromedial/mediolateral conditions. Although the 0.7 cm difference of length between the two sides might seem negligible, as supported by the similar efficacy for inducing illusions between conditions, our results still encourage testing further the impact of changing the shapes and sizes of vibrators, like a 2.5 x 2.5 cm surface of contact. However, the particular directions of illusions reported with the lateromedial condition cannot be solely explained by the smaller surface of contact. Why did the lateromedial orientation resulted in more frequent illusions with an ulnar deviation component (cf. Table 3), whereas the mediolateral condition, having the exact same surface of contact and position, caused infrequent ulnar or radial components?

The only differential factor between the mediolateral and lateromedial conditions was the rotational direction of the mass (clockwise, counterclockwise). Shifting between clockwise and counterclockwise rotations likely changed the way the motor ‘hit’ underlying structures, as supported by orbital analyses of eccentric rotating mass systems [21, 22]. The resulting asymmetric elliptical orbits could change the angle and locus of maximal energy transfer between the vibrator and the participant in a way that favored the lateral side of the vibrator for the lateromedial condition, and the medial side for the mediolateral. Similarly, the distoproximal and proximodistal conditions would have resulted in a slight change in energy transmission favoring either the distal or the proximal part of the vibrator. From an anatomical standpoint, the effect of changing the rotational direction for the distoproximal and proximodistal conditions is negligible since both the proximal and distal sides of the vibrator are in contact with the same tendons. However, the situation differs for the mediolateral and lateromedial conditions. Indeed, the distal tendon of flexor carpi radialis is located slightly lateral to the wrist’s center, near other flexor muscles (palmaris longus, flexor digitorum superficialis, flexor digitorium profundus). Therefore, placing the vibrator at the center of the palmar surface of the wrist like we did and using the lateromedial orientation (highest energy transfer toward the lateral side of the vibrator) would be more effective to target these wrist flexors and radial deviators. Our results did find that illusions with an ulnar deviation component (especially combined to extension) were significantly more frequent during lateromedial versus the other orientations, hence supporting the idea of a higher activation of flexor carpi radialis' muscle spindles. One would expect that the same applies to the mediolateral orientation and illusions with a radial deviation component (i.e. higher energy transfer on the medial side of the vibrator toward wrist flexors and ulnar deviators), however, the distal tendon of flexor carpi ulnaris is located farther from the center with its insertion near the fifth finger on the pisiform bone [31, 32]. In line, we did not find higher occurrence of radial deviations when using the mediolateral or any other orientation. Still, perceptions of deviations and other complex multiplanar or multi-joint movements were sometimes perceived by our participants, possibly because vibration spread to nearby muscles (e.g. pronator quadratus). Of note, the higher variability of directions with the lateromedial orientation is probably not caused by more vague and weaker illusions that could have challenged the ability of the participants to identify the precise direction. Indeed, illusions were of similar clearness and speed and amplitude between all four orientations. This further supports that the lateromedial orientation is effective in vibrating tendons and creating kinesthetic illusions, but that this particular orientation preferentially stimulates different tendons (like the flexor carpi radialis) compared to the other orientations. Thus, our results support that changing the rotational direction of the eccentric mass and the vibrator’s shape and surface of contact influence the effects of vibration, especially when targeting complex anatomical areas comprising several tendons unequally distributed. Although such results might only apply to vibrators of similar shape and design (i.e. eccentric rotating mass) than ours, they nonetheless highlight the importance of better considering the impact of these underrecognized technological and anatomical aspects of muscle tendon vibration. However, other factors unexplored by the present work should be considered in future studies, like the contribution of skin mechanoreceptors to kinesthetic illusions [33, 34] since eccentric rotating mass vibrators also move back and forth parallel to the skin with a main direction of skin displacement likely shifting between the four tested conditions. ”

Comment #2: Furthermore, it is unclear why data analysis is based on the median rather than the mean which can be very misleading. For example, though no significance is reported, the results in Table 2 seem to suggest that participants perceived different VAS speed/amplitude in all four conditions (e.g., 3.70 vs 4.00 vs 3.43 vs 4.30), which would be counter-intuitive given the mechanism of the ERM motor. If there are specific reasons to report median values, they must be stated. In the paper cited for the SKIP scoring system (ref. 19), the comparison was made based on mean values.

- **Answer:** To clarify, significance statistics (e.g. p values) and descriptive statistics (e.g. median) are different concepts. We did not make comparisons based on median values. Rather, all individual data was imported in SPSS to realize statistical tests of contrasts such as the Wilcoxon signed-rank and McNemar tests. The median and interquartile showed in Table 2 were only used to describe the central tendency (median) and spread (IQR) of the data. Similarly, comparisons between healthy and stroke participants in Ref. 19 were not made based on the mean, but on individual data. Means and SDs presented in their paper were used to summarize and illustrate group data, not to confirm or refute differences between groups.

Further, significant contrasts between conditions of orientations (PD, DP, ML, LM rotation) or between sides are in fact clearly identified in Table 2 using superscript letters; if there is no superscript letter then no difference was found by the statistical tests. So, conversely to the Reviewer’s comment, Table 2 does not suggest that VAS scores were different between conditions. However, we agree with the Reviewer that the text describing results from Table 2 and Table 3 could have been clearer, especially for VAS findings. This has been corrected in the revised manuscript (lines 237-278).

The only significant contrasts were found for scores of direction between the LM and DP, and between the LM and PD (cf. ‘a’ and ‘b’ superscript letters in Table 2 and lines 258-262). Of note, a Bonferroni correction was applied to limit the risk of Type I errors since 4 conditions of orientation were compared (i.e. 6 pair-wise comparisons = 0.05 alpha level of significance divided by 6 = p < 0.008333), so contrasts between LM and DP/PD were below p < 0.00833. These methodological choices, their justification and description were presented in the manuscript (lines 209-234, and Table’s 2 legend).

As for the choice of median & IRQ instead of the mean & SD, we simply followed best practices from reference publications in the field of statistics for health applications [2.1; 2.2]. The mean and SD are appropriate representations of the central tendency and spread when data are normally distributed and based on continuous scales. These conditions were not met in the present work, since ordinal and dichotomous scores were used to measure illusory perceptions and knowing that the data was not normally distributed (cf. lines 210-211). There is an important risk of using the mean and SD with non-normal / skewed data or in the presence of outliers, as demonstrated in reference books [2.1; 2.2] articles [2.3; 2.4] and website dedicated to statistics [2.5; 2.6; 2.7]:

References :

[2.1] Portney, L. G., & Watkins, M. P. (2009). *Foundations of Clinical Research applications to practice* (3rd ed.). Pearson Education, Inc.

[2.2] Domholdt, E. (2005). *Rehabiltation Research – Principles and Applications* (3rd ed.). Elsevier Inc.

[2.3] Sainani, K. L. (2012). Dealing with non-normal data. *Pm&r*, *4*(12), 1001-1005.

[2.4] Mishra, P., Pandey, C. M., Singh, U., Gupta, A., Sahu, C., & Keshri, A. (2019). Descriptive statistics and normality tests for statistical data. Annals of cardiac anaesthesia, 22(1), 67-72.

[2.5]

[https://statistics.laerd.com/statistical-guides/measures-central-tendency-mean-mode-median.php](https://statistics.laerd.com/statistical-guides/measures-central-tendency-mean-mode-median.php )

[2.6] <https://statistics.laerd.com/statistical-guides/measures-central-tendency-mean-mode-median-faqs.php>

[2.7] <https://statistics.laerd.com/statistical-guides/measures-of-spread-range-quartiles.php>

Nevertheless, no consensus has been reached by statisticians on the ‘best’ choice between median and mean for ordinal scores having a higher level of precision (such as the VAS or Likert scale – cf. [2.6]); the choice often relies more on personal preferences which could explain why authors in Ref. 19 chose to show results as means and SD in their Figure 3. But despite having used means/SD, they selected the same statistical tests to verify their hypothesis than the ones used here. Of note, only the corresponding author from the present manuscript was involved in Ref. 19 as a PhD trainee; the final choices for how to present data thus relied on preferences from a different team of researcher compared to the present work. Based on the best-practices from reference publications cited above, we stand behind our choice of presenting group data for SKIP scores as median and IQR.

Changes made to the manuscript relating to Comment #2:

**Results**

“As shown in Table 2, SKIP clearness scores and VAS scores of speed and amplitude were not significantly different for pooled and non-pooled data between the four conditions of rotational direction (all p>0.05). Clearness of kinesthetic illusions was stable across the four conditions, with participants mostly rating “*moderately clear and precise*” movements. Direction scores showed significantly lower scores (i.e. any other direction than the expected wrist extension) for the lateromedial versus the proximodistal (p=0.0002) and distoproximal conditinos (p=0.008). VAS data also pointed toward low to moderate sensations of speed and amplitude, with median scores between 2.58-4.53 and 25^th^-75^th^ quartiles ranging between 1.37-6.52.”

Comment #3: It is also confusing how movement perceived by the participant is “attributed by the evaluator to one of twelve possible perceptions at the wrist.” There is no description of how this is performed, and if I understand correctly, the information mainly comes from verbal description, since the participant needs to “give specific details on the perceived movement.” If this is the case, the results would be highly unreliable because normal participants are not trained to specify enough details for the evaluator to clearly categorize as many as 12 motions—the results in Table 3 would contain too much error for further analysis. The authors are suggested to reexamine the study design and the collected data to perform solid analysis for the research question.

- **Answer:** We agree that more information is needed to confirm that movements perceived by the participants were correctly identified by the evaluator. Our study is not the first to reliably report complex sensory perceptions such as multi-joint or even drawing-like movements successfully measured in previous publications [3.1]. It is true that the general public lacks knowledge about the anatomical names of joint movements. They are however able to show what they felt using their wrist when the verbal description was unclear. The main assessor was a trained physiotherapist with all the required experience and knowledge to easily recognize the perceived sensations. We clarified the procedure used for collecting the directions of illusions in the manuscript (lines 175-179; 219-220; 254-255).

References:

[3.1] Thyrion, C., & Roll, J. P. (2010). Predicting any arm movement feedback to induce three-dimensional illusory movements in humans. *J Neurophysiol*, *104*(2), 949-959. <https://doi.org/10.1152/jn.00025.2010>

jn.00025.2010 [pii]

Changes made to the manuscript in response to Comment #3:

**Methods**

“To quantify and compare the subjective perceptions of movement reported by the participants, movements were attributed by the evaluator to one of twelve possible perceptions at the wrist (extension, extension with ulnar deviation, ulnar deviation, flexion with ulnar deviation, flexion, flexion with radial deviation, radial deviation, extension with radial deviation, supination, pronation, circumduction, no movement perceived).”

“Then, frequency of occurrence (in %) for each movement perceived were calculated and compared across conditions according to qualitative description of participants’ perceptions. Combined movements were calculated based on the sum of each frequency where we observed a combination of 2 movements (i.e. extension and ulnar deviation, flexion and radial deviation) and complex movements (i.e. supination, pronation and circumduction). The same pattern was used to obtain totals within a specific component (e.g. all perceptions that included an extension component).”

**Results**

“Frequency of occurrence for the 12 perceptions of wrist movements reported by the participants were calculated and listed in Table 3. Rate of success for eliciting a kinesthetic illusion, independently of its direction, was high (95.4-100%) for the four conditions of rotational orientation. The most frequent movement perceived by the participants across the four conditions was wrist extension, ranging between 57.7-76.8 % of trials. This frequency of wrist extension occurrence was similar between conditions, except for one pair-wise comparison resulting in a significantly lowest frequency for lateromedial compared to distoproximal orientations (p=0.009). Multi-planar or multi-joint movement illusions were reported in about 18% of all trials. Occurrence for these combined/complex illusions was highest for the lateromedial orientation (26.1%). Lateromedial rotation caused illusory movements with an ulnar deviation component in 17.7% of trials, which seemed higher than proximodistal (6.3% - p=0.049), distoproximal (7.2% - p=0.016) and mediolateral (5.4% - p=0.021) conditions, but did not reach the significance level after applying the Bonferroni correction. Conversely, results with the lateromedial condition showed that illusions involving an ulnar deviation component were significantly more frequent than illusions with a radial deviation component (p=0.004). No other difference was found between conditions and directions of illusory perceptions.”

Comment #4: [52] It is unclear why vibration should be abbreviated as VIB.

- **Answer**: To improve consistency, we removed the use of the abbreviation VIB in the main manuscript. Changes are marked in red throughout the text.

Comment #5: [57–59] I suppose “a few studies” refer to ref. 9–12, and in this case, the period after “retraining sensorimotor control” should be colon.

- **Answer:** We corrected this passage for an improved clarity and grammar:

**“**A few studies assessed the clinical utility of kinesthetic illusions in the context of sensorimotor disorders [9-12]. In stroke, vibration-induced illusions were found as less clear on the paretic limb compared to the non-paretic limb and healthy counterparts [9, 10], supporting its validity to identify proprioceptive deficits. Also, a repeated application of vibration showed promise as a proprioceptive-based intervention to improve upper-limb sensorimotor control [11, 12].”

Comment #6: [65] Throughout the manuscript, the term “clear and precise” is used. While this appears to be two separate aspects, it is treated as a single property (at least indicated by the results). I suggest avoiding such ambiguity unless the authors have clear definitions of “clearness” and “precision” in the context of kinesthetic illusion. Also, avoid formats such as “clear & precise” or “clear/precise.”

- **Answer:** The SKIP’s measure of clearness and precision was in fact imported from previous studies on kinesthetic illusions, including important work by JP Roll, one of the most renown researcher in the field [3.1]. The terms ‘clear and precise’ are used as a single property, simply to give more examples to help the participant rates the level of clarity perceived. The choices offered by our experimenter were the same than those previously used by Roll’s team [3.1], as part as the SKIP (Ref. 19):
  - Perfectly clear and precise, like a real perception
  - Moderately clear and precise
  - Vague and not precise

This way of measuring the vividness of the illusory perceptions was in fact adapted by Roll’s team from psychophysical scales developed for mental imagery, including the well-accepted Betts’ questionnaire [6.1]. We are not aware of any publication having tried to demonstrate that clearness and precision are different aspects that should be studied separately, although there are other publications that simply asked the participants about the ‘strength’ of the illusion without including the terms clearness or precision [6.2]. There is currently no consensus on which terms should be preferred to rate the illusion’s vividness, nor is there any specific definitions for the different terms used in the literature. In our study, we preferred to remain coherent with our previous and current research, as well as publications from renown experts in vibration and kinesthetic illusion. We made a few changes throughout the manuscript in response to the Reviewer’s comment, most often by simply removing “precise” when not describing the SKIP measures, and by changing “&” or “/” by “and”.

References:
[6.1] **Sheehan PW.** A shortened form of Betts’ questionnaire upon mental imagery. *J Clin Psychol* 23: 386 –389, 1967.

[6.2] Schofield, J. S., Dawson, M. R., Carey, J. P., & Hebert, J. S. (2015). Characterizing the effects of amplitude, frequency and limb position on vibration induced movement illusions: Implications in sensory-motor rehabilitation. *Technology and Health Care*, *23*(2), 129-141. <https://doi.org/10.3233/thc-140879>

Comment #7: [70] The semicolon should be a colon.

- **Answer:** We have corrected the syntax of the sentence.

Comment #8: [81–84] This text should be enhanced with discussion over the mechanism of vibration motor as well as how vibration direction is changed if the motor is rotated by 90°. Note that the rotational direction of the mass (equivalent to 180° rotation) is irrelevant to the induced vibration.

- **Answer:** As explained in depth in response the Comment #1, we made several changes to the manuscript to better present and discuss how ERM works and how the rotational direction explains some of our findings (cf. for example lines 78-100).

Comment #9: [95–96] This claim is inappropriate.

- **Answer:** The reviewer is right, and we apologize for this mistake. The sentence was deleted (cf. lines 115-116).

Comment #10: [99–102] The grammatic subject of this sentence should be “participants.”

- **Answer**: We corrected this passage following the Reviewer’s suggestion (lines 119-120).

Comment #11: [117] “Then,…”

- **Answer**: We thank the Reviewer for their suggestion that removes unnecessary words and improve the sentence’s clarity (cf. lines 138-139).

Comment #12: [142–146] The past tense suddenly changes to the present tense.

- **Answer:** The corrections were made in the manuscript (lines 163-167).

Comment #13: [168] Ref. 19 is the work of the corresponding author, so it is inappropriate to use the term “as recommended,” as if the authors are citing an external source.11. [191–196] How this is performed must be clarified.

- **Answer:** The Reviewer is right, we removed the term “as recommended”. The method for rating clearness and precision is described in lines 167-171.

Comment #14: [191–201] This should be part of the experimental procedure.

- **Answer:** We remove this passage and rather described how the directions of illusions were recorded by the evaluator in the methods (lines 173-180).

Comment #15: [Table 2] Report mean±STD.

- **Answer**: As explained in response to Comment #2, we prefer not to report those results as mean and SD since our data does not meet the statistical requirements (normal distribution, continuous scales).

Comment #16: [Table 3] Most motions have a frequency lower than 3%, which is just one or two trials and much more susceptible to error. If anything, only the last five rows should be shown, but the authors must prove the assessment was performed in a reliable way. For example, I don’t see how it is possible to separate the perception of extension/flexion from ulnar/radial deviation based on verbal description.

- **Answer:** As explained in response to Comment #3, identifying the illusion’s direction was not really an issue in our study. The participants were allowed to show what they perceived with their wrist after each trial if verbal description was unclear. For a trained physiotherapist, recognizing a wrist extension/flexion from an ulnar/radial deviation is not difficult. The fact that complex perceptions such as pronation or bi-planar movements were rarely reported does not automatically question their validity and reliability. The same applies to ‘No illusion’ which was also rarely reported (0-6.4%) but not necessarily at higher risk of error. We prefer to remain transparent with our results by reporting all perceptions, even those that were less frequent, since it highlights the important intra- and inter-individual variability of kinesthetic perceptions. As explained in the Discussion (lines 384-394), such variability can be seen as a strength or a weakness, depending on how vibration is used and the desired outcomes. And we are not convinced that only keeping the last five row would be better, since their scores are less specific. For example, the “Total with extension component” in fact includes any trial that had at least a component of wrist extension (thus including Extension + UD and Extension + RD illusions).
